# Effect of heat stress on vital and hematobiochemical parameters of healthy dogs

**DOI:** 10.14202/vetworld.2022.722-727

**Published:** 2022-03-25

**Authors:** Oyebisi Mistura Azeez, Folashade Helen Olaifa, Adakole Sylvanus Adah, Afisu Basiru, Ganiu Jimoh Akorede, Hauwa Moturayo Ambali, Kolawole Yusuf Suleiman, Fatima Sanusi, Mashood Bolaji

**Affiliations:** 1 Department of Veterinary Physiology and Biochemistry, University of Ilorin, Ilorin, Nigeria; 2 Department of Veterinary Pharmacology and Toxicology, University of Ilorin, Ilorin, Nigeria; 3 Department of Veterinary Medicine, University of Ilorin, Ilorin, Nigeria; 4 Department of Veterinary Pathology, University of Ilorin, Ilorin, Nigeria

**Keywords:** blood, dog, environment, heat index

## Abstract

**Background and Aim:**

Heat stress is a major challenge for animals, impairing their welfare and performance. This study aimed to determine the effect of heat stress on the vital and hematobiochemical parameters of healthy dogs.

**Materials and Methods:**

The experimental subjects comprised 10 dogs, encompassing seven males and three non-pregnant females between 2 and 3 years of age. Ambient temperature (AT) and relative humidity (RH) were recorded 2 hourly during the day and the temperature humidity index was calculated. Vital parameters [i.e., rectal temperature (RT), respiratory rate, and heart rate (HR)] were assessed and blood was collected from each dog daily for hematobiochemical analysis.

**Results:**

The RT (38.5±0.2°C) of dogs exposed to high AT and high RH (HA/HR) conditions was significantly (p<0.05) higher than that of dogs exposed to HA and low RH (LR) conditions (37.2±0.11°C). Under HA/HR conditions, packed cell volume, hemoglobin concentrations, and white blood cell counts were significantly lower than those of the same dogs exposed to HA/LR conditions. Conversely, under HA/HR conditions, the lymphocyte, monocyte, eosinophil, alanine aminotransferase, aspartate aminotransferase, and cortisol values were significantly higher (p<0.05) than the values obtained in dogs exposed to HA/LR conditions. Meanwhile, the alkaline phosphatase, urea, and glucose levels were significantly lower (p<0.05) in dogs exposed to HA/HR conditions.

**Conclusion:**

The exposure of healthy dogs to HA/HR conditions induced heat stress, which may have an adverse effect on their immune status, thereby affecting their health and welfare.

## Introduction

In animals, heat stress occurs when the heat load is greater than the animal’s capacity to lose heat [1]. In other words, heat stress is exacerbated when the physiological systems of the body fail to regulate the body temperature within the normal range. High ambient temperature (HAT) and relative humidity (RH) with increased solar radiation and low air movement are all factors that contribute to heat stress [2].

Heat stress is a threat to animal well-being [3] and may lead to decline in the health, immunity, and productivity of animals, including dogs used for breeding, security, consumption, hunting, and research purposes [4-6]. The vital parameters of rectal temperature (RT), respiratory rate (RR), and heart rate (HR), in addition to hematobiochemical parameters are useful for evaluating heat stress and diagnosing stress-related diseases in animals [7]. The determination of these parameters permits an assessment of many metabolic and physiological processes and variations in these parameters provide helpful diagnostic information.

To date, there has been no extensive report on the effect of heat stress on the vital and hematobiochemical parameters of healthy dogs in the study area. This study aimed to determine the effect of heat stress on the vital and hematobiochemical parameters of healthy dogs.

## Materials and Methods

### Ethical approval

The study was carried out humanely in accordance with the guidelines, governing the welfare of research animals by the University of Ilorin, and as approved by the Ethics Research Committee of the University of Ilorin, Ilorin, Nigeria, under the permit number UERC/FVM/024/2021.

### Study period and location

The study was conducted from January to April 2019. The study was carried out at the dog kennel of the Department of Veterinary Physiology and Biochemistry, University of Ilorin, Ilorin, Nigeria, which is a transitional zone, between the forest and the guinea savannah regions of Nigeria (Lat. 8°30’ and 8°50’N and Log. 4°20’ and 4°35’E). The total annual rainfall ranges from 800 mm to 1200 mm in the NW and 1000 mm to 1500 mm in SE.

### Experimental animals and management

Ten Nigerian local breed of dogs comprising seven males and three non-pregnant females, aged between 2 and 3 years with an average weight of 15.9±0.9 kg served as experimental subjects. They were obtained from Ilorin (Lat. 8°30’ and 8°50’N and Log. 4°20’ and 4°35’E), Nigeria. During the study period, they were fed with homemade bland diet at 08:00 h and 18:30 h, daily and were given water *ad libitum*. Their body score was 5 out of 9-point scale. Routine medical examination including hemoparasite screening was carried out on all the dogs. The dogs were vaccinated against rabies, canine distemper, and leptospirosis. It was ensured that the dogs were calm before sampling.

### Experimental design

All the dogs (n=10) served as both control and test subjects. Each dog was housed individually in a standard kennel (36’×24’) at the Department of Veterinary Physiology and Biochemistry, University of Ilorin, Ilorin, Nigeria. Under the test condition, the dogs were exposed to HA and high RH (HR) during the peak of the hot season (April). In January, as a control condition, the dogs were exposed to HA and low RH (LR). During the study period, for each dog, vital parameters (i.e., RT, RR, and HR) were recorded 2 hourly basis and blood samples were collected. During both the test and control conditions, all vital parameters and blood samples were measured and collected daily for 2 weeks, respectively.

### Measurement of meteorological parameters

AT was recorded at the experimental site using a wet- and dry-bulb thermometer (Brannan, England). Throughout the study period, RH was obtained using Osmond’s hygrometric table (Narindra Scientific Industries, Haryana, India). THI was determined using the formula below:

THI=(1.8AT+32)–(0.55–0.0055RH/100) (1.8AT–26) [8].

Where;

THI=Temperature humidity index

RH=Relative humidity.

### Measurement of vital parameters

RT was measured according to the methods described by Cugmas *et al*. [9]. Briefly, RT was recorded using a digital thermometer (Electron Thermometer, COCET, Kangfu Medical Equipment, Zhejiang Yueqing, China). The thermometer was first turned on and allowed to calibrate, then advanced slowly into the dog’s rectum (approximately 1” deep) and held firmly in place to ensure reading accuracy and ease of removal. The thermometer was held in place for 2 min until a beeping sound was heard, indicating that the reading had completed. RR was measured according to the method described by Lopedote *et al*. [10]. Briefly, RR was assessed by observing and counting the number of respiratory abdominal movements for 1 min; the rate was measured in cycles per minute. HR was recorded according to the method reported by Katayama *et al*. [11]. Briefly, HR was recorded through auscultation of the heart, assessed by placing a stethoscope (Sprague, Rappaport Type Stethoscope, England) between the second and fifth rib on the dog’s left side and counting the number of beats per minute. All dogs were handled by familiar and experienced clinicians.

### Blood sample collection

Blood (10 mL) was aseptically collected from each dog daily by cephalic venipuncture using a 5 mL syringe and 23-gauge sterile needle. Blood samples were collected into two 5 mL syringes: The sample from one syringe was placed in a sample bottle with anticoagulant for hematological analysis and the sample from the other syringe was placed in a sample bottle without anticoagulant to extract serum samples for biochemical analysis.

### Determination of hematological parameters

Red blood cell and white blood cell (WBC) counts were determined using a hemocytometer (Merck KGaA, Darmstadt, Germany) [12]. Packed cell volume (PCV) was estimated using the microhematocrit method [12], whereas the hemoglobin (Hb) concentration was estimated using the cyanmethemoglobin method [13]. Differential leukocyte counts were determined as described by Dacie and Lewis [14]. The mean corpuscular volume (MCV), mean corpuscular Hb (MCH), and MCH concentration (MCHC) were calculated as described by Esievo [15].

### Determination of biochemical parameters

The blood samples were allowed to clot at room temperature (24-26ºC) for 30 min and then centrifuged at 3000× *g* for 15 min. Sera were carefully harvested into labeled vials, then immediately analyzed. The concentrations of creatinine, urea, total protein, bilirubin, albumin, glucose, aspartate aminotransferase (AST), alanine aminotransferase (ALT), alkaline phosphatase (ALP), and electrolytes such as sodium (Na^+^), potassium (K^+^), chloride (Cl^−^), calcium (Ca^2+^), and bicarbonate (HCO_3_−) were measured using commercial test kits (Agappe, India) and a digital ultraviolet spectrophotometer (PerkinElmer AAS 400, USA). Cortisol was determined using a commercial radioimmunoassay kit (Coat-a-count Cortisol, Siemens Medical Solution Diagnostics, Los Angeles, CA), according to the manufacturer’s protocol [16].

### Statistical analysis

The obtained data were expressed as mean±standard errors of the mean and analyzed using a Wilcoxon matched pair signed-rank test. The relationship between meteorological parameters and vital parameters was evaluated using Pearson’s correlation analysis. GraphPad Prism 5.0 (San Diego, California, USA) was used to conduct all the analyses. Statistical significance was set at p<0.05.

## Results

Table-1 shows the meteorological parameters of the environment during the study period. Under control conditions (HA/LR), the dogs were exposed to HA (33.3±1.2°C) and LR (46.6±8.6%). Under test conditions (HA/HR), the dogs were exposed to HA (32.2±0.9°C) and HR (61.4±5.6%). RH was significantly (p<0.05) lower during the HA/LR conditions than during the HA/HR conditions. The THI during exposure to HA/LR and HA/HR was 85.5±3.3 and 80.5±2.9, respectively.

**Table-1:** Meteorological parameters during the study period.

Condition	Ambient temperature (°C)	Relative humidity (%)	Temperature humidity index
HA/HR	32.2±0.9	61.4±5.6^a^	80.5±2.9
HA/LR	33.3±1.2	46.6±8.6^b^	85.5±3.3

^a,b^Values with different superscripts on the same column are significantly (p<0.05) different. HA/HR=High ambient temperature/high relative humidity, HA/LR=High ambient temperature/low relative humidity

Table-2 shows the vital parameters of the local healthy dogs exposed to heat stress. The RT (38.5±0.2°C) recorded from dogs exposed to HA/HR was significantly higher (p<0.05) than the RT recorded from dogs exposed to HA/LR (37.2±0.11°C). There was no significant difference between the RR recorded in dogs exposed to HA/LR (45.8±2.5 cycles/min) and HA/HR (42.9±4.6 cycles/min). Furthermore, there was no significant difference between the HR of dogs exposed to HA/LR (114.1±1.6 beats/min) and HA/HR (145±6.5 beats/min).

**Table-2:** Vital parameters of healthy dogs exposed to heat stress.

Parameters	HA/HR (minimum-maximum)	HA/LR (minimum-maximum)
Rectal temperature (°C)	38.5±0.2^a^ (37.7-39.2)	37.2±0.11^b^ (36.3-38.6)
Respiratory rate (cycles/minute)	42.9±4.6 (29-64)	45.8±2.5 (28-64)
Heart rate (beats/minute)	145±6.5 (115.3-170)	114.1±1.6 (84-160)

^a,b^Values with different superscripts on the same row are significantly (p<0.05) different. HA/HR=High ambient temperature/high relative humidity, HA/LR=High ambient temperature/low relative humidity

The results of the hematobiochemical measurements are presented in Tables-3 and 4. PCV (25.8±0.9%) and Hb (7.9±0.2 g/dL) recorded in dogs exposed to HA/HR were significantly lower than those (37.8±1.1% and 12.7±0.4 g/dL, respectively) recorded in dogs exposed to HA/LR. Compared to dogs exposed to HA/LR, the WBC count was significantly lower in dogs exposed to HA/HR. Lymphocyte, monocyte, and eosinophil counts were significantly higher (p<0.05) in dogs exposed to HA/HR, compared to those of dogs exposed to HA/LR. Moreover, the MCV, MCH, and MCHC values were numerically lower during exposure to HA/HR; however, these differences were statistically non-significant. Dogs exposed to HA/HR had significantly higher ALT, AST, and cortisol levels (p<0.05), and significantly lower (p<0.05) ALP, urea, and glucose levels, compared to dogs exposed to HA/LR.

**Table-3:** Hematological responses of healthy dogs (n=10) to heat stress.

Hematological parameters	HA/HR (minimum-maximum)	HA/LR (minimum-maximum)
PCV (%)	25.8±0.9^a^ (13-31)	37.8±1.1^b^ (24-48)
Hb (g/dL)	7.9±0.2^a^ (4.4-10.1)	12.7±0.4^b^ (8-17)
RBC (×10^12^/L)	4.2±0.1 (2.2-5.2)	5.4±0.2 (3-7)
WBC (×10^9^/L)	9.3±0.8^a^ (5.1-20)	11.4±0.5^b^ (8-17)
Neutrophil (×10^9^/L)	4.9±2.2^a^ (3.5-7.4)	8.3±0.6^b^ (3.5-14.5)
Lymphocyte (×10^9^/L)	4.1±2.2^a^ (1.8-5.6)	1.8±0.2^b^ (0.2-4.9)
Monocyte (×10^9^/L)	1.7±0.1^a^ (1-3)	0.5±0.1^b^ (0-1.4)
Eosinophils (×10^9^/L)	1±0.0^a^ (1-1)	0.4±0.1^b^ (0-1.7)
Basophil (×10^9^/L)	0.0±0.0 (0-0)	0.0±0.0 (0-0)
MCV (fl)	61.4±0.8 (50-68)	70.3±0.8 (63.3-77)
MCH (pg)	19±0.2 (15.9-20.4)	22.8±0.5 (21-26.2)
MCHC (g/dL)	30.9±0.3 (26.7-34.1)	33.7±0.3 (29.4-35.8)
Platelet (×10^9^/L)	291±16.8^a^ (148-431)	462.4±39.7^b^ (211-621)

^a,b^Values with different superscripts on the same row are significantly (p<0.05) different. PCV=Packed cell volume, Hb=Hemoglobin, RBC=Red blood cell, WBC=White blood cell, MCV=Mean corpuscular volume, MCH=Mean corpuscular hemoglobin, MCHC=Mean corpuscular hemoglobin concentration, HA/HR=High ambient temperature/high relative humidity, HA/LR=High ambient temperature/low relative humidity

**Table-4:** Biochemical responses of healthy dogs (n=10) to heat stress.

Biochemical parameters	HA/HR (minimum-maximum)	HA/LR (minimum-maximum)
Potassium ion (mmoL)	4.6±0.3 (3.5-5.9)	4.5±0.1 (3-6)
Chloride ion (mg/dL)	86.8±1.6^a^ (78-95)	117±1.5^b^ (100-132)
Bicarbonate ion (mmoL)	22.7±0.8 (18-26)	22±0.8 (16-32)
ALT (U/L)	31±8.3^a^ (14-95)	24±4.3^b^ (5-95)
AST (U/L)	30.8±3.9^a^ (11-48)	12.6±0.5^b^ (7-20)
ALP (U/L)	27.8±4^a^ (13-49)	51.3±7^b^ (7-100)
Creatinine (mg/dL)	0.8±4.2 (0.06-1)	1±0.01 (0.4-1.8)
Urea (mmol/L)	2.8±0.2^a^ (2-4.1)	20±2.8^b^ (8-28)
Total bilirubin (mg/dL)	2.2±0.3 (1.2-3.4)	1±0.2 (0.5-1.7)
Total protein (g/L)	61.6±4.2 (50-90)	59±2.1 (23-76)
Glucose (mmoL/L)	3.3±0.3^a^ (2-4.8)	5.4±0.2^b^ (4.2-6.6)
Albumin (g/L)	23.6±3.2 (22-36)	25.4±1.5 (10-30)
Calcium (mmol/L)	2.1±0.2 (1.3-3)	2.6±0.1 (2.3-2.9)
Cortisol (µmol/L)	5.5±1.2^a^ (0.2-11)	0.02±0.003^b^ (0.03-0.01)

^a,b^Values with different superscripts on the same row are significantly (p<0.05) different. AST=Aspartate aminotransferase, ALT=Alanine aminotransferase, ALP=Alkaline phosphatase, HA/HR=High ambient temperature/high relative humidity, HA/LR=High ambient temperature/low relative humidity

Table-5 illustrates the relationship between meteorological parameters and the dogs’ vital parameters. There was a significant positive relationship between AT with RT (r=0.7535, p<0.01) and RR (r=0.6852, p<0.05) during exposure to HA/HR. Similarly, there was a significant positive relationship between RH with RT (r=0.8905, p<0.01) and RR (r=0.6329, p<0.01) during exposure to HA/HR.

**Table-5:** Relationship between meteorological and vital parameters of healthy dogs.

Correlated parameters	HA/HR (r)	HA/LR (r)
Ambient temperature and;		
Rectal temperature	0.7535**	0.2429
Respiratory rate	0.6852**	0.6400**
Heart rate	0.0090	−0.4002
Relative humidity and;		
Rectal temperature	0.8905***	0.6787**
Respiratory rate	0.6972*	0.8998***
Heart rate	0.1255	0.1396
Temperature-humidity index and;		
Rectal temperature	0.6548**	0.1027
Respiratory rate	0.6329**	0.4583
Heart rate	−0.3198	−0.5204*

* Significant (p<0.05)

** Highly significant (p<0.01)

*** Most highly significant (p<0.001), HA/HR=High ambient temperature/high relative humidity, HA/LR=High ambient temperature/low relative humidity

## Discussion

The climatic conditions of HA/HR have been established as a major cause of heat stress in animals [17]. Furthermore, the THI values recorded during this study were higher than the reported thermally comfortable value of 70 [18]. Thus, the combined effect of HA, RH, and THI may induce heat loss difficulty in dogs [18]. The exposure of dogs to HA/LR may not be stressful as dogs can successfully adapt to their environments [19]; thus, they may be subjected to less stress.

The notable increase in the RT of dogs exposed to HA/HR indicates a physiological response to heat stress exposure. However, due to the HR, the dogs may not have been able to adequately lose their accumulated body heat since they mostly regulate their temperature through panting [20]. Under HA/HR conditions, panting becomes progressively less effective for dissipating excessive body heat, thus dogs’ body cooling mechanism becomes inadequate. Nonetheless, exposing the dogs to HA/LR did not significantly increase their RT, since they can dissipate approximately 70% of their total body heat through radiation and convection from body surfaces, which is conveniently effective at LR [21]. Animal breed, coat type/length, and coat color influence canine temperature [22]; however, in this study, the same group of dogs served as both the control and test animals, thereby eliminating these effects on their temperature.

In this study, the decreased PCV and Hb values concur with the findings of Odo *et al*. [23] who noted decreased PCB and Hb in rats subjected to heat stress; this anemia-like finding may be attributed to hemodilution as a result of the increased consumption of fluid for evaporative cooling during HA/HR conditions [24]. Similarly, the lower values of MCV, MCH, and MCHC observed in this study were in tandem with the values obtained by Ukwueze *et al*. [25], which may be due to stress; this was confirmed by the observed increased lymphocyte, neutrophil, and eosinophil counts [26]. Moreover, the lower observed WBC counts indicate that dogs may have a poor immune status when exposed to HA/HR conditions.

High cortisol level was observed in response to heat stress [27]. Circulating cortisol has been reported to be a very sensitive index of heat stress due to poor tolerance to severe climates. It has been reported to be outside the normal range of dogs suffering from heat stroke [28]. In this study, the dogs’ cortisol was significantly higher when exposed to HA/HR, indicating that they were experiencing heat stress. The increased levels of ALT and AST also indicate that the dogs were responding to the effects of heat stress [29]. Moreover, the reduced level of glucose in dogs exposed to HA/HR agrees with the findings of Kanter [30] who indicated that heat stress induces hypoglycemia. Under HA/HR conditions, metabolism is higher and there is an increased dilation of blood vessels; both factors lead to increased absorption of insulin, which induces hypoglycemia. Furthermore, the lower concentrations of chloride ion and urea noted in this study indicate that the dogs may have been overhydrated due to increased water consumption [31].

The positive relationship between AT with RT and RR and between RH with RT and RR was highly significant in dogs exposed to HA/HR conditions. This finding indicates that as AT or RH increase, RT and RR increase consecutively, altogether denoting that the dogs were more responsive to changes in these environmental parameters and thus were subjected to stress. One limitation of this study is that the influence of cloud cover and wind speed was not considered with respect to influencing heat stress in dogs under both conditions.

## Conclusion

The exposure of healthy dogs to HA/HR conditions induced heat stress and may have an adverse effect on their immune status, thereby affecting their health and overall welfare. The effect of antioxidants on dogs exposed to such stressful conditions could be an avenue for future research.

## Authors’ Contributions

AOM, OFH, AAS, and BA: Designed the study, statistical analysis, and manuscript writing and editing. AGJ, AHM, SKY, and SF: Collected and tabulated data. BM: Assisted in analyzing the result and manuscript writing. All authors read and approved the final manuscript.

## References

[1] Habeeb A.A.M, Osman S.F, Gad A.E (2020). Signs of heat stress and some steps to reduce the negative effects on animals. GSC Adv. Res. Rev.

[2] Herbut P, Angrecka S, Walczak J (2018). Environmental parameters to assessing of heat stress in dairy cattle-a review. Int. J. Biometeorol.

[3] Sejian V, Devaraj C, Krishnan G, Bagath M, Chauhan S.S, Suganthi R.U, Fonseca V.F.C, Konig S, Gaughan J.B, Dunshea F.R, Bhatta R (2021). Heat stress and goat welfare:Adaptation and production considerations. Animals (Basel).

[4] Ajala O.O, Fayemi O.E, Oyeyemi M.O (2012). Some reproductive indices of Nigerian local bitches in Ibadan, Nigeria. Niger. J. Physiol. Sci.

[5] Njoga U.J, Ajibo F.E, Njoga E.O (2020). The use of contraceptive for the control of stray dog population and spread of rabies virus in Nigeria. Anim. Res. Int.

[6] Bappah M.N, Chom N.D, Lawal M, Bada A.A, Saidu T.M (2021). Evaluation of vertebral heart score and cardiac sphericity in apparently normal dogs. Iran. J. Vet. Surg.

[7] Nie D, Culi J, Zhao N, Chen M (2020). Haematological and serum biochemical reference values in Chinese water deer (*Hydroptes inermis*):A preliminary study. BMC Vet. Res.

[8] Habeeb A.A, Gad A.E, El-Tarabany A.A, Aha M.A.A (2018). Negative effects of heat stress on growth and milk production of farm animals. J. Anim. Husbandry Dairy Sci.

[9] Cugmas B, Šušterič P, Gorenjec N.R, Plavec T (2020). Comparison between rectal and body surface temperature in dogs by the calibrated infrared thermometer. Vet. Anim. Sci.

[10] Lopedote M, Valentini S, Musella V, Vilar J.M, Spinella G (2020). Changes in pulse rate, respiratory rate and rectal temperature in working dogs before and after three different field trials. Animals (Basel).

[11] Katayama M, Kubo T, Mogi K, Ikeda K, Nagasawa M, Kikusui T (2016). Heart rate variability predicts the emotional state in dogs. Behav. Proc.

[12] Schalm O.W, Jain N.C, Carroll E.J (1975). Normal haematology of horse and donkey. In:Veterinary Haematology.

[13] Baker F.J, Silverton R.E (1985). Introduction to Medical Laboratory Technology.

[14] Dacie J.V, Lewis S.M (1991). Practical Haematology.

[15] Esievo K.A.N (2017). Veterinary Clinical Pathology.

[16] Gold A.J, Langlois D.K, Refsal K.R (2016). Evaluation of basal serum or plasma cortisol concentrations for diagnosis of hypoadrenocorticism in dogs. J. Vet. Intern. Med.

[17] Oke O.E, Uyanga V.A, Iyasere O.S, Oke F.O, Majekodunmi B.C, Logunleko M.O, Abiona J.A, Nwosu E.U, Abioja M.O, Daramola J.O, Onagbesan O.M (2021). Environmental stress and livestock productivity in hot-humid tropics:Alleviation and future perspectives. J. Therm. Biol.

[18] Xiong Y, Meng Q, Gao J, Tang X, Zhang H (2017). Effect of relative humidity on animal health and welfare. J. Integr. Agric.

[19] Lundholm B (1976). Domestic animals in arid ecosystems. Ecol. Bull.

[20] McKinley M, Trevaks D, Weissenborn F, McAllen R (2017). Interaction between thermoregulation and osmoregulation in domestic animals. Rev. Bras. Zootec.

[21] Bruchim Y, Horowitz M, Aroch I (2017). Pathophysiology of heatstroke in dogs revisited. Temperature.

[22] Kwon C.J, Brundage C.M (2021). Quantifying body surface temperature differences in canine coat types using infrared thermography. J. Therm. Biol.

[23] Odo R, Onoja S.O, Osuagwu C.O (2019). Impact of heat stress on blood and serum biochemistry parameters in rats. Not. Sci. Biol.

[24] Morar D, Ciulan V, Simiz F, Mot I.H, Vaduva C (2018). Effect of heat stress on haematological parameters of dairy cows. Lucr Stiint Ser Med. Vet.

[25] Ukwueze C, Akpan E.S, Ezeokonkwo R.C, Nwosu C.I, Anene B.M (2020). Haematology, oxidative stress and electrolyte alterations in puppies with canine parvoviral enteritis. Iraqi J. Vet. Sci.

[26] Hickman D.L (2017). Evaluation of neutrophil:Lymphocyte ratio as an indicator of chronic distress in the laboratory mouse. Lab. Anim.

[27] Sundman A.S, Van Poucke E, Holm A.C.S, Roth L.S.V (2019). Long-term stress levels are synchronized in dogs and their owners. Sci. Rep.

[28] Assia E, Epstein Y, Magazanik A, Shapiro Y, Sohar E (1989). Plasma cortisol levels in experimental heat stroke in dogs. Int. J. Biometeorol.

[29] Al-Shammarai A.S.K, Al-Hashimi A.D.M, Rabeea A.H.M (2019). Impact of thermal stress on health signs, hormone levels, haematological and biochemical parameters of police dogs in Iraq. Adv. Anim. Vet. Sci.

[30] Kanter G.S (1959). Cause of hypoglycemia in dogs exposed to heat. Am. J. Physiol.

[31] Goggs R, De Rosa S, Fletcher D.J (2017). Electrolyte disturbances are associated with non-survival in dogs a multivariable analysis. Front. Vet. Sci.

